# The Story of the Insane from Year to Year

**Published:** 1904-09-24

**Authors:** 


### THE STORY OF THE INSANE FROM YEAR TO YEAR.

Sixteenth Annual Series.

### HANTS COUNTY ASYLUM.

On the first of January there were in residence 1,138 patients, and by the 31st December the number had fallen to 1,090, but this decrease was unfortunately not caused by any diminution in the numbers of the insane, but was owing to a rearrangement of districts whereby Bournemouth no longer sent its patients to the County Asylum. In fact, the increase among the county cases proper has been 38 in six months, so that the asylum will soon be full again. There were 206 first admissions, 34 readmissions, 72 discharged recovered, 9 discharged relieved, 93 discharged not improved, and 114 died. The average number resident was 1,096, and the total number of cases under treatment was 1,378. Calculated on the admissions the recoveries stand at 30 per cent, and calculated on the average number resident the deaths stand at 10·4 per cent. The former percentage is lower than the average of the last forty years, and the latter is higher than in the previous year, and is attributed to the deaths from colitis. Of the deaths, 33 were due to cerebral and spinal diseases, 12 to consumption, 10 to other lung diseases, and 20 to colitis. The latter disease broke out in January, and 86 patients were stricken. It is remarkable that one nurse contracted the disease and died of it. Dr. Worthington thinks that overcrowding is one cause of the disease, and in extreme cases it may be, but we doubt whether it ever exists to such an extent in English asylums as to account for an outbreak. That it may tend to the spread of the disease is probable enough. Much more likely is the other cause named, the disturbance of the soil in the neighbourhood of old drains, but even this does not quite explain the fact that the insane are attacked out of all proportion to the sane. At the Hants Asylum something like 6 per cent. of the patients were attacked, and perhaps less than 1 per cent. of the staff

The committee "desire to express their high appreciation of the services of Dr. Worthington." The weekly rate of maintenance is 9s. 11d. per head.

### INSANE HOSPITAL AT WORCESTER, MASSACHUSETTS.

Here there were 1,116 patients in residence at the beginning of the year, but 12 of these, all women, are classed as "habitual drunkards;" and at the end of the year the total number in residence was 1,184. The total number under treatment during the year was 1,706, and the average number resident was 1,125. There were 624 admissions, 137 discharged recovered, 75 discharged much improved, and 117 died. Intemperance in drink, either alone or in conjunction with other causes, accounted for 214 of the year's admissions, and heredity for 50, while 86 were of unknown cause. Among the causes of death we note that consumption figures 16 times, and pneumonia and other lung diseases 54 times. Dr. Quinby, the medical superintendent, has nine assistant physicians, and one of these is a lady. With such a staff a vast amount of work ought to be done, and we gather from Dr. Quinby's words that it is done. He says: "Not so very long ago it was the complaint in regard to our insane hospitals that they were nothing but large boarding-houses, and that little if anything was done towards the treatment and cure of the inmates. If even this was true of any one of them, which I am unwilling to acknowledge, they can now, I am sure, fairly claim to have gotten beyond the boarding-house stage, and to have become in reality hospitals." And Dr. Quinby gives the comfortable assurance that, "as a consequence of changed methods, the whole medical atmosphere of the institution has changed, and a very superior class of young physicians has been attracted." A new pathological building and various other improvements are in progress.

### LUNATIC ASYLUM, JAMAICA.

At the beginning of the year this asylum contained 863 patients and at the end of the year the number had risen to 959. There were 202 first admissions, 24 readmissions, 76 recoveries, 51 discharged not improved, and 54 deaths. The average number resident was 915, and the total number under treatment was 1,089. The asylum is overcrowded, but we read that "during the year it was found possible to fit up and occupy a small ward of the buildings so long unfinished." This permitted the transfer of 60 male patients, but was no relief to the female wards and unless immediate steps are taken it will be necessary to stop the admission of patients. Who is responsible for this state of affairs? The weekly cost of maintenance was 6s. 1d. per head.

### LONDON COUNTY ASYLUM AT BEXLEY HEATH.

The number resident at the beginning of the year was 2,[illegible]51, and at the end of the year it was 2,083. There were 483 first admissions, 36 readmissions, 180 discharged recovered, 28 discharged relieved, 29 not improved, and 250 died. The average number resident was 2,079, and the total number under treatment during the year was 2,570. The recoveries were 39 92 per cent. of the admissions, and the deaths were 12 02 per cent. of the average number resident. Of the year's deaths 103 were caused by cerebral and spinal diseases, general paralysis alone accounting for 61 of this number, 17 by consumption, 17 by other lung diseases, 11 by colitis, dysentery, or enteritis, 2 by enteric fever, and 1 by small-pox. Of the year's admissions various moral causes are said to account for the insanity in 40 instances, and among the physical causes intemperance in drink accounts for 103, heredity for 211, and previous attacks for 92. During the year female nurses have taken the place of male attendants in the men's infirmary wards, and Dr. Stansfield is so much pleased with the innovation that he thinks when the plans of a new asylum are being considered the matter should receive attention; but we doubt, whether the majority of medical superintendents are as yet converted to the principle, and although Dr. Stansfield says it has "added materially to the comfort, the general well-being, and the successful treatment of the patients," we are not specifically told why these good points have followed the innovation, and why they might not have been obtained with good male nurses. When speaking of the colitis, Dr. Stansfield says that "its incidence does not appear to have been materially affected by the rigid isolation which has been practised, particularly on the male side, where every case of diarrhœa has at once been transferred to the East Villa and has remained there at least 14 days after the complete disappearance of all symptoms. In the female division, where the facilities for such rigid isolation did not exist, and where the cases had to be treated in an infirmary ward, the incidence of the cases is wonderfully similar to that in the male division." It is by careful observations such as these that we may one day hope to arrive at some definite conclusions as to the cause of this disease. At present there is much conflict of opinion. The committee "desire to place on record their appreciation of the zeal which Dr. Stansfield, the other officers of the asylum, and the general staff continue to display in the discharge of their duties." The weekly cost per head is 11s. 5d.

